# Maternal Nutritional Adherence and Second-Trimester Amniotic-Fluid Metabolomic Profiles: An Exploratory Study

**DOI:** 10.3390/medicina62071217

**Published:** 2026-06-23

**Authors:** Giulia Gaudiello, Jacopo Troisi, Laura Sarno, Maurizio Guida, Ludovica Niccolini, Carmen Ziello, Davide De Vita

**Affiliations:** 1 Department of Public Health, University of Naples Federico II, 80131 Naples, Italy; niccoliniludovica@gmail.com (L.N.); carmenziello99@gmail.com (C.Z.); 2 Presidio Ospedaliero Santa Maria Della Speranza, 84091 Battipaglia, Italy; d.devita@aslsalerno.it; 3 Department of Medicine, Surgery and Dentistry “Scuola Medica Salernitana”, University of Salerno, 84081 Baronissi, Italy; jtroisi@unisa.it; 4 Theoreo srl, Spinoff Company of the University of Salerno, 84090 Montecorvino Pugliano, Italy; 5 European Institute of Metabolomics Foundation (EIM), Via G. Puccini, 84081 Baronissi, Italy; 6 Department of Neuroscience, Reproductive Sciences and Dentistry, University of Naples Federico II, 80131 Naples, Italy; laura.sarno@unina.it

**Keywords:** fetal programming, maternal nutrition, amniotic fluid, metabolomics, Developmental Origins of Health and Disease (DOHaD), pregnancy

## Abstract

*Background and Objectives*: Maternal nutrition during pregnancy has been associated with fetal development and long-term health trajectories through mechanisms potentially involving epigenetic and metabolic programming. However, the molecular mediators linking dietary quality to fetal biochemical profiles remain poorly characterized. This exploratory pilot study aimed to investigate associations between maternal nutritional adequacy and the amniotic-fluid metabolomic profile during the second trimester. *Materials and Methods*: In this prospective study, AF samples from 41 pregnant women undergoing second-trimester amniocentesis were analyzed using gas chromatography–mass spectrometry (GC-MS). Nutritional status was assessed via the FIGO Nutrition Checklist. Subjects were divided into two groups based on dietary adequacy (FIGO Score >5 vs. ≤5). Multivariate analysis (PLS-DA, VIP scores, volcano plots) and pathway enrichment were performed to identify discriminatory metabolites. *Results*: Exploratory metabolomic analysis suggested differences between the two nutritional groups. Several candidate metabolites, including stearic acid, lactic acid, proline, and scyllo-inositol, contributed to the observed differences between groups. These features may provide preliminary hypotheses regarding energy-, amino acid-, and lipid-related biochemical pathways. *Conclusions*: Maternal dietary quality was associated with differences in the amniotic-fluid metabolomic profile. These preliminary findings support further investigation of amniotic-fluid metabolomics as a potential tool for studying the fetal biochemical environment.

## 1. Introduction

Human development is a complex and dynamic process shaped by the continuous interplay between genetic inheritance and environmental exposures. While the genome provides the foundational blueprint for growth and function, it is the environment—particularly during critical windows of development—that modulates this blueprint, giving rise to individual variability in health and disease outcomes. This concept has been increasingly acknowledged through the lens of epigenetics, which comprises heritable modifications in gene expression that do not involve alterations in the underlying DNA sequence but are instead mediated by mechanisms such as DNA methylation, histone modification, and non-coding RNAs [1].

Among all periods of life, the first 1000 days, encompassing conception through the first two postnatal years, are considered the most plastic and vulnerable to environmental modulation. This window is not only critical for organogenesis and neurodevelopment but also establishes long-term physiological setpoints, thereby determining susceptibility or resilience to chronic diseases in later life [2]. This paradigm shift, now central to the Developmental Origins of Health and Disease (DOHaD) hypothesis, emphasizes that adult pathologies—such as cardiovascular disease, obesity, diabetes, and neuropsychiatric disorders—may have their roots in fetal programming, a process by which early-life exposures leave lasting physiological imprints [3].

Fetal programming is particularly influenced by the intrauterine environment, shaped not only by maternal genetic and physiological states but also by modifiable factors, such as nutrition, stress, infection, and exposure to endocrine-disrupting chemicals. Among these, maternal nutrition is one of the most potent and actionable determinants. A diet poor in macro- and micronutrients or characterized by high caloric intake, excessive fat, or deficiency in critical vitamins and minerals can adversely influence placental function, inflammatory tone, and epigenetic programming, ultimately affecting fetal growth trajectories [4].

The profound role of maternal diet in fetal development is underscored by epidemiological evidence linking prenatal nutritional status to the risk of developing non-communicable diseases. For instance, maternal obesity and excessive gestational weight gain are associated with increased risks of Large for Gestational Age (LGA) neonates, childhood obesity, glucose metabolism disturbances, and cardiometabolic disorders in later life [5].

Mechanistically, this programming is thought to involve epigenetic modifications of key metabolic genes during embryogenesis, setting long-term patterns in energy balance, immune function, and neurodevelopment. Yet, the characterization of these changes at a molecular level has remained a challenge—until the emergence of metabolomics, a powerful high-throughput approach that captures the end-products of gene-environment interactions.

Metabolomics is the comprehensive analysis of low molecular weight metabolites in biological systems. It provides an integrated snapshot of the physiological state of a cell, tissue, or organism, reflecting not only genetic inputs but also environmental influences, including diet, drugs, and lifestyle exposures [6,7]. Unlike genomics or transcriptomics, which provide potential insights, metabolomics captures the real-time phenotype and is therefore uniquely positioned to monitor subtle biochemical shifts that underlie developmental plasticity.

The amniotic fluid (AF) represents an ideal matrix for studying the fetal metabolome. With its dual origin—maternal through transplacental transport and fetal through urine production and skin transudation—it acts as a medium that reflects both maternal inputs and fetal metabolic activity. From the 17th week of gestation onward, AF becomes predominantly fetal in origin, offering a window into in utero metabolic programming [8].

Emerging studies have demonstrated that maternal diet can influence the chemical composition of AF. For example, Mennella et al. [9] demonstrated that the ingestion of garlic derivatives by pregnant women significantly altered the odor profile of AF, suggesting the potential for dietary metabolites to cross the placental barrier and influence the intrauterine environment. However, such observations have rarely been contextualized within a high-resolution metabolic framework.

In recent years, AF metabolomics has gained attention as a promising tool for identifying biomarkers of fetal health, early predictors of congenital anomalies, and risk stratification for conditions such as intrauterine growth restriction, preterm birth, and neurodevelopmental disorders [10]. Yet, its utility in understanding the impact of maternal diet on fetal metabolic trajectories remains largely unexplored.

Through GC-MS and LC-MS platforms, AF metabolomics can reveal disruptions in key pathways such as fatty acid metabolism, amino acid biosynthesis, oxidative stress, and energy metabolism. For example, altered levels of proline, lactic acid, or stearic acid have been associated with a range of pathophysiological conditions, including asthma, diabetes, and obesity—all of which are increasingly recognized as having roots in early-life exposures [11].

Moreover, metabolomic analysis not only enables the identification of disease-associated biomarkers but also helps in unraveling metabolic networks and pathway perturbations, paving the way for precision nutrition strategies during pregnancy.

Despite mounting evidence supporting the role of maternal diet in fetal programming, there remains a significant gap in understanding the biochemical mediators that translate maternal dietary patterns into fetal metabolic phenotypes. Most current research focuses on maternal serum or cord blood analysis, while direct fetal compartment analysis remains rare.

This study aims to bridge that gap by analyzing the metabolomic composition of amniotic fluid in relation to maternal dietary quality, assessed through the FIGO Nutrition Checklist—a validated tool developed by the International Federation of Gynecology and Obstetrics to assess adherence to periconceptional nutritional guidelines.

This study aimed to explore whether maternal nutritional adequacy, assessed using the FIGO Nutrition Checklist, was associated with differences in the metabolomic composition of amniotic fluid collected during clinically indicated second-trimester amniocentesis. The analysis was designed as an exploratory investigation to identify candidate metabolic features and pathways potentially related to maternal nutritional status, generating hypotheses for future longitudinal and mechanistic studies.

## 2. Materials and Methods

### 2.1. Study Design and Sample Collection

This prospective, observational study was conducted at the Obstetrics and Gynecology Unit of the University of Naples “Federico II”, located in Campania, Southern Italy.

Eligible participants were pregnant women undergoing clinically indicated second-trimester amniocentesis at the Obstetrics and Gynecology Unit of the University of Naples “Federico II”. Inclusion criteria were singleton pregnancy, maternal age ≥ 18 years, availability of amniotic-fluid sample collected during the clinically indicated procedure, completion of the FIGO Nutrition Checklist, availability of essential clinical and anthropometric data, and written informed consent for the use of anonymized clinical and biological data for research purposes.

A total of 41 pregnant women were enrolled during the second trimester of gestation at the time of clinically indicated amniocentesis. Gestational age at amniotic-fluid collection was recorded for each participant and expressed in completed weeks of gestation.

Women diagnosed with fetal chromosomal abnormalities, or who declined to provide informed consent, were excluded from participation.

All enrolled participants completed a structured medical history and underwent an obstetric evaluation within the week preceding amniocentesis. On the day of the procedure, after a 12-h fasting period, amniotic fluid (AF) was collected following standard protocols, without extracting additional volumes solely for research purposes. Clinical and anthropometric data—including age, BMI, and gestational weight gain—were recorded.

Nutritional status was assessed using the FIGO Nutrition Checklist, which provides a structured evaluation of adherence to key nutritional recommendations during pregnancy. For the purpose of this exploratory analysis, participants were stratified according to their total checklist score into two groups: adequate nutritional adherence, defined as a FIGO score > 5, and lower nutritional adherence, defined as a FIGO score ≤ 5. This threshold was defined a priori to distinguish women with higher versus lower adherence to FIGO nutritional recommendations. However, it was used as an exploratory stratification criterion and should not be interpreted as a validated diagnostic cut-off for nutritional inadequacy.

The study protocol was approved by the institutional Ethics Committee (approval n° 483/21), and all participants provided written informed consent in accordance with the Declaration of Helsinki.

### 2.2. Cytokine Quantification

Cytokine levels were quantified in a subset of 37 amniotic-fluid samples, including 28 samples from women with higher nutritional adherence (FIGO Nutrition Checklist score > 5) and all 9 samples from women with lower nutritional adherence (FIGO Nutrition Checklist score ≤ 5). Cytokine profiling was performed in samples with sufficient residual amniotic-fluid volume available after clinical testing and metabolomic analysis. Because cytokine quantification was not available for the entire cohort, cytokine–metabolite correlation analyses were considered exploratory and interpreted cautiously. Cytokines were measured using a multiplex bead-based immunoassay (Bio-Plex Pro™ Human Cytokine Assay, Bio-Rad Laboratories, Hercules, CA, USA), following the manufacturer’s instructions.

Cytokine and inflammatory-marker concentrations were quantified according to the manufacturer’s instructions and analytical specifications. Lower limits of detection were those reported by the manufacturer for each analyte in the assay documentation. Values below the lower limit of detection were imputed using the analyte-specific limit of detection divided by the square root of two (LOD/√2) before statistical analysis. Missing cytokine values unrelated to detection limits were handled by pairwise deletion, so that each metabolite–cytokine correlation was calculated using only samples with available values for both variables. Samples were analyzed in duplicate, and duplicate measurements were inspected for analytical consistency before downstream analysis. Because cytokine profiling was performed as an exploratory subset analysis, laboratory-specific inter-assay coefficients of variation were not calculated; analytical precision was assessed according to the manufacturer’s quality-control criteria and duplicate measurement consistency.

The panel included TNF-α, IL-1β, IL-17E, GM-CSF, IL-18, NGAL, IL-5, IL-10, MCP-1, YKL-40, IFN-γ, IL-6, and TGF-β. Fluorescence intensity was measured with a dedicated Luminex™ system and expressed as pg/mL. Each result was anonymized using numerical patient codes and stored securely within the institutional biobank.

### 2.3. Metabolomic Analysis

Untargeted metabolomic profiling of AF was carried out via Gas Chromatography–Mass Spectrometry (GC-MS). Sample preparation, including metabolite extraction, purification, and derivatization, was performed using the MetaboPrep GC Kit (Theoreo S.r.l., Montecorvino Pugliano, Italy), as previously described by Troisi et al. [12].

Briefly, 50 µL of each AF sample was transferred into microcentrifuge tubes and mixed with an alcohol-based extraction buffer containing 2-isopropyl malic acid as the internal standard. After 30 min of orbital shaking (1250 rpm) and 30 min of sonication, samples were centrifuged at 16,000 rpm for 5 min at 4 °C. The supernatant (200 µL) was added to a second tube containing purification reagent, briefly vortexed (30 s, 1250 rpm), and centrifuged again under the same conditions. The clarified supernatant (175 µL) was transferred to glass vials, frozen, and subsequently lyophilized overnight.

Derivatization was carried out in two phases at room temperature: initially, 50 µL of methoxylamine hydrochloride in pyridine was added and shaken (90 min, 1200 rpm), followed by 25 µL of BSTFA-based silylation solution, mixed for an additional 90 min. Derivatized samples were centrifuged once more (5 min, 16,000 rpm, 4 °C), and 1.8 µL of the final solution was injected into a Shimadzu GCMS-2010SE system in split mode (5:1).

Chromatographic separation was achieved using a CP-Sil 8 CB capillary column (30 m × 0.25 mm, 1.00 µm film thickness, Agilent Technologies (Santa Clara, CA, USA)), with helium as the carrier gas at a constant linear velocity of 39 cm/s. The oven temperature protocol began at 100 °C (1 min hold), increased to 320 °C at a rate of 6 °C/min, and was maintained for 4 min, for a total run time of 40 min. The mass spectrometer was operated in electron impact ionization mode (70 eV), scanning over an *m*/*z* range of 35–600, with a scan rate of 3333 amu/sec and a solvent delay of 4.5 min.

Samples were analyzed in batches of 25, each including 4 quality controls: (i) Reagent blank (2 µL of hexane), (ii) Mixed standard (50 reference metabolites), (iii) Technical replicate of a randomly selected sample, (iv) Pooled sample control.

Quality acceptance criteria included: (i) Absence of signal in blanks, (ii) Peak area consistency of the standard mix (±10% of expected), (iii) ≤15% coefficient of variation in the top 100 peaks of replicate samples, (iv) Pooled sample clustering within 5% of prior reference PCA models.

### 2.4. Data Processing and Metabolite Identification

Metabolites detected in fewer than 80% of the samples were excluded. Raw chromatographic data were processed using GCMS Solution v2.72 (Shimadzu, Kyoto, Japan) for peak detection and deconvolution.

Metabolite annotation was performed according to the Metabolomics Standards Initiative (MSI) criteria. Detected features were annotated at MSI level 2 when spectral similarity against the NIST-14 mass spectral library was supported by linear retention-index agreement, using a retention-index tolerance < 50 and a minimum spectral match threshold of 85%. Features that could not be reliably assigned were retained as unknown features and classified as MSI level 4. The metabolites reported as VIP discriminant features were identified at MSI level 1 by comparison with an internal library of authentic analytical standards analyzed under the same chromatographic and mass-spectrometric conditions. Chromatographic alignment and missing value imputation were performed using the MetaboPredict platform (Theoreo. Montecorvino Pugliano, Italy), which employs parametric time warping via the ptw R package (ver. 1.9.13) [13].

### 2.5. Statistical Analysis

Clinical data were analyzed with MedCalc Software (ver. 14.8.1.0) (Ostend, Belgium). Normal distribution of variables was assessed using the Kolmogorov–Smirnov test. Group comparisons were evaluated using two-tailed, unpaired *t*-tests, with *p* < 0.05 considered statistically significant.

For metabolomic data, chromatographic peak areas were log-transformed and autoscaled (mean-centered and scaled to standard deviation). Samples were arranged in a matrix format (samples × metabolites) for multivariate modeling.

Partial Least Squares–Discriminant Analysis (PLS-DA) was used to explore group separation. Variable importance in projection (VIP) scores were calculated, with a significance threshold set at ≥1.5. To assess model robustness, a permutation test with 2000 random label reassignments was performed.

PLS-DA model performance was evaluated using cross-validation and permutation testing. The following parameters were reported: R^2^Y, Q^2^, accuracy, sensitivity, specificity, and balanced accuracy. Because of the imbalance between groups, balanced accuracy was considered particularly relevant for model interpretation. A permutation test based on 2000 random class-label assignments was used to assess whether the observed model performance exceeded that expected by chance. When available, CV-ANOVA was also reported as an additional assessment of model reliability. Additionally, a volcano plot analysis was used to visualize statistically and biologically relevant metabolites, plotting −log10 (*p*-value) against log_2_ fold change (FC). Metabolites with large fold changes and low *p*-values appeared in the upper right or left quadrants, denoting high discriminatory power.

For metabolite–cytokine correlation analysis, Pearson correlation coefficients were calculated between candidate dysregulated metabolites and measured cytokines. Given the number of tested correlations, *p*-values were adjusted for multiple comparisons using the Benjamini–Hochberg false discovery rate procedure. Correlations were interpreted as statistically supported only when they remained significant after FDR correction.

Pathway analysis was conducted using the onlline MetPa tool (accessed 10 May 2025) [14] and the Human Metabolome Database (HMDB) [15]. Over-representation was calculated via Fisher’s Exact test (α = 0.05) to explore candidate biochemical pathways represented by the metabolites contributing to group differences. Given the limited and imbalanced sample size, all supervised multivariate analyses, VIP-based metabolite ranking, volcano plot analysis, and pathway enrichment results were interpreted as exploratory and hypothesis-generating rather than confirmatory.

## 3. Results

### 3.1. Maternal Characteristics and Nutritional Stratification

A total of 41 pregnant women were included in the analysis: 32 in the higher nutritional adherence group (FIGO Nutrition Checklist score > 5) and 9 in the lower nutritional adherence group (FIGO Nutrition Checklist score ≤ 5).

All participants underwent second-trimester amniocentesis as part of routine clinical care. Specifically, the clinical indication for amniocentesis was recorded for each participant. Indications included advanced maternal age, positive first-trimester screening, increased risk at combined test, abnormal ultrasound findings, family history, and maternal request. No additional amniotic-fluid volume was collected for research purposes beyond that required for the clinically indicated procedure. The distribution of clinical indications for amniocentesis was comparable between the higher and lower nutritional adherence groups, suggesting no major imbalance in referral indications between groups. The demographic and anthropometric characteristics of the study population are reported in Table 1. Maternal age did not differ significantly between the two groups, with a mean age of 33.46 ± 5.95 years in the higher nutritional adherence group and 35.50 ± 7.13 years in the lower nutritional adherence group.

Because the FIGO threshold was used for exploratory stratification, group comparisons were interpreted as associations with higher versus lower nutritional adherence rather than as diagnostic categories of nutritional adequacy or inadequacy. Pre-pregnancy and current body weight, as well as body mass index (BMI), tended to be higher in Group 2, although these differences did not reach statistical significance. Notably, the average gestational weight gain was also greater among women in the inadequate nutrition group. Gestational age at amniotic-fluid collection was comparable between the two nutritional groups. In the higher nutritional adherence group, the mean gestational age was 125.25 ± 11.69 days, corresponding to 17.89 ± 1.67 weeks, whereas in the lower nutritional adherence group it was 126.18 ± 15.45 days, corresponding to 18.03 ± 2.21 weeks. No statistically significant difference was observed between groups (Welch *t*-test, *p* = 0.812; Mann–Whitney test, *p* = 0.758).

### 3.2. Metabolomic Profiles and Group Separation

Untargeted metabolomic analysis of amniotic fluid was performed to explore whether maternal nutritional adequacy was associated with detectable differences in the fetal biochemical profile. The PLS-DA score plot suggested partial group separation according to maternal dietary adherence. However, because of the limited sample size and the imbalance between groups, this supervised model was interpreted cautiously and used primarily as an exploratory visualization of the data structure.

The PLS-DA model was built using 4 latent components and evaluated using a cross-validation scheme. The model showed R^2^Y = 0.771, Q^2^ = 0.563, and an overall accuracy of 0.831. Because the cohort was imbalanced, with 32 participants in the higher nutritional adherence group and 9 in the lower nutritional adherence group, the majority-class baseline accuracy was 0.780. Therefore, the observed accuracy showed only a modest improvement over the majority-class baseline and was interpreted cautiously. Based on the confusion matrix, sensitivity was 0.867, specificity was 0.611, and balanced accuracy was 0.739. The 2000-permutation test yielded a permutation *p*-value of 0.041, suggesting that the observed discrimination exceeded that expected from random class assignment.

### 3.3. Identification of Discriminatory Metabolites

The most influential metabolites driving the observed separation were identified through Variable Importance in Projection (VIP) scores, with a threshold of VIP > 1.5. A complementary volcano plot analysis was also performed to identify statistically significant changes (*p* < 0.05) with a fold-change threshold (log_2_FC > 2).

The combined exploratory analyses identified a set of metabolites that contributed most strongly to the observed group separation and may represent candidate metabolic features associated with lower nutritional adherence.

These metabolites were more abundant in the inadequate nutrition group; however, given the exploratory nature of the analysis and the small group size, this pattern should be interpreted cautiously and requires confirmation in larger cohorts.

**Figure 1:**
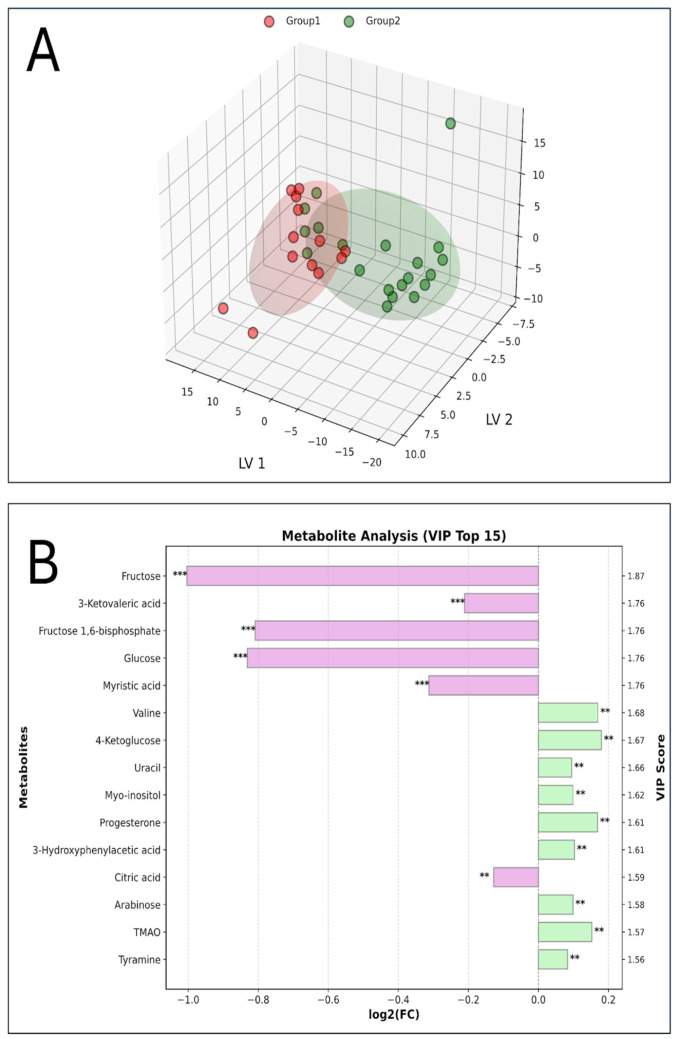
Exploratory multivariate and univariate analyses of metabolomic profiles in amniotic-fluid samples according to maternal nutritional adherence. (**A**) Three-dimensional Partial Least Squares–Discriminant Analysis (3D-PLS-DA) score plot showing partial exploratory separation between samples from the higher nutritional adherence group (FIGO Nutrition Checklist score > 5; red) and the lower nutritional adherence group (FIGO Nutrition Checklist score ≤ 5; green). Given the limited and imbalanced sample size, the PLS-DA plot should be interpreted as an exploratory visualization rather than evidence of robust group discrimination. (**B**) Variable Importance in Projection (VIP) scores for the top 15 candidate metabolites contributing to the exploratory separation, plotted against their corresponding log_2_ fold change (FC). Metabolites relatively more abundant in the lower nutritional adherence group are shown in purple, while those relatively more abundant in the higher nutritional adherence group are shown in green. Asterisks indicate nominal levels of statistical significance: ** *p* < 0.01 and *** *p* < 0.001. Fructose, 3-keto-valeric acid, and fructose-1,6-bisphosphate were among the highest-ranking candidate metabolites.

The MSI level 1 metabolites contributing to the exploratory group separation included stearic acid, lactic acid, proline, scyllo-inositol, mandelic acid, phenylacetic acid, 2,4-dihydroxybutanoic acid, ribose, benzoic acid, and threitol (Figure 1).

### 3.4. Pathway Enrichment Analysis

Pathway enrichment analysis was performed to explore the possible biological context of the candidate metabolites associated with lower nutritional adherence. The highest-ranking pathways included phenylalanine metabolism, pyruvate metabolism, glycolysis/gluconeogenesis, biosynthesis of unsaturated fatty acids, arginine and proline metabolism, and aminoacyl-tRNA biosynthesis.

**Figure 2:**
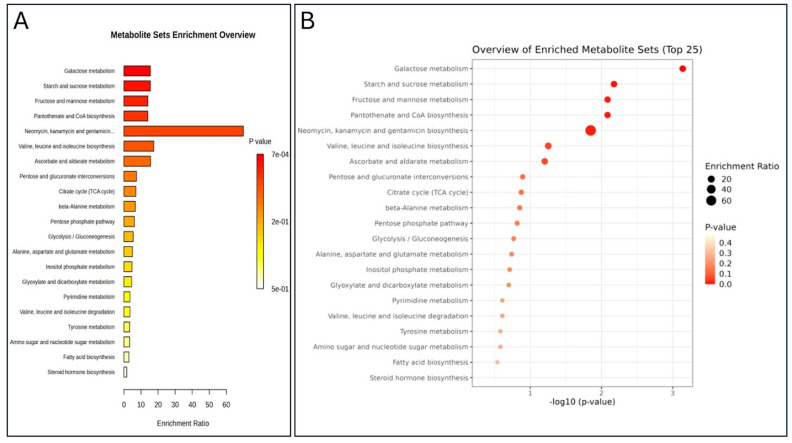
Exploratory pathway enrichment analysis of candidate metabolites differing between nutritional groups. (**A**) The bar plot and bubble plot show the highest-ranking pathways according to enrichment ratio and Holm-adjusted *p*-values. Because adjusted *p*-values did not pass conventional significance thresholds, these pathways should be interpreted as exploratory and hypothesis-generating rather than statistically confirmed. The results may provide biologically plausible directions for future validation studies. Panel (**B**) displays the broader set of highest-ranking pathways, whereas the Section 3 focuses on selected candidate pathways considered biologically relevant to the study question.

However, after Holm correction, adjusted *p*-values ranged from 0.05 to 0.22 and did not meet conventional thresholds for statistical significance. Therefore, these results should be interpreted as exploratory and hypothesis-generating rather than as evidence of significantly enriched pathways. Nevertheless, the identified pathways may provide biologically plausible directions for future studies investigating maternal nutrition, fetal metabolism, and developmental programming (Figure 2).

### 3.5. Correlations Between Dysregulated Metabolites and Inflammatory Cytokines in Amniotic Fluid

Cytokine profiling was available for 37 amniotic-fluid samples, including 28 samples from the FIGO > 5 group and all 9 samples from the FIGO ≤ 5 group. Therefore, metabolite–cytokine correlation analyses were performed as exploratory analyses. Correlations were evaluated to investigate potential associations between candidate dysregulated metabolites and the intra-amniotic inflammatory milieu, without implying causal relationships.

To explore potential associations between candidate dysregulated metabolites and the intra-amniotic inflammatory milieu, correlation analysis was performed between 11 metabolites and 13 cytokines/inflammatory markers. Because this analysis involved multiple comparisons, *p*-values were adjusted using the Benjamini–Hochberg false discovery rate procedure.

**Figure 3:**
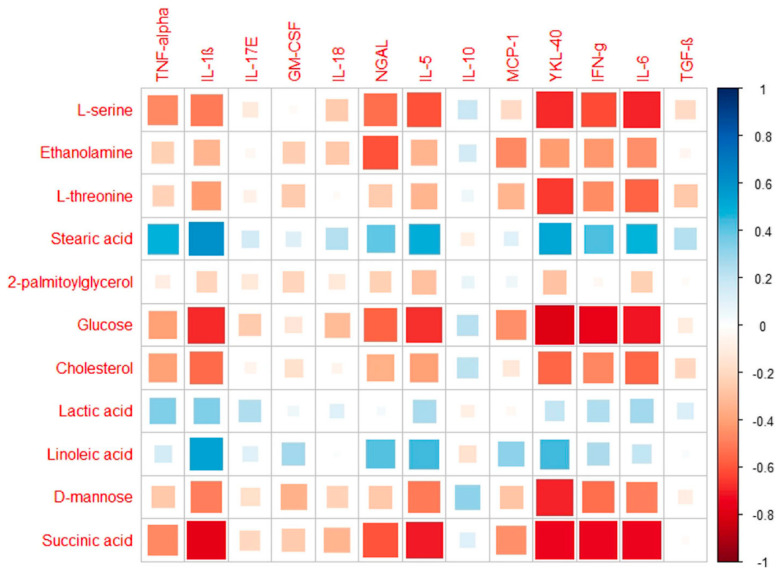
Correlation matrix between dysregulated metabolites and inflammatory cytokines in amniotic fluid. Heatmap showing the Pearson correlation coefficients between 11 significantly altered metabolites and 13 immune-inflammatory markers measured in amniotic fluid samples. Color intensity represents the strength and direction of the correlation: blue indicates positive correlations, red indicates negative correlations, while the size of each square is proportional to the correlation coefficient (ranging from −1 to +1). Notably, succinic acid and D-mannose exhibited positive associations with pro-inflammatory cytokines such as TNF-α, IL-6, IL-1β, and MCP-1, whereas stearic acid and 2-palmitoylglycerol showed inverse relationships with IL-1β, IL-10, and IL-17E. This heatmap provides an exploratory visualization of possible associations between candidate metabolites and cytokines in amniotic fluid. Given the number of tested correlations and the subset-based cytokine analysis, these patterns should be interpreted cautiously and require validation in larger cohorts.

Several nominal correlations were observed between selected metabolites and cytokines. However, after correction for multiple testing, these associations were interpreted cautiously. Therefore, the heatmap should be considered an exploratory visualization of possible metabolite–cytokine relationships rather than confirmatory evidence of immunometabolic interactions (Figure 3).

Among the notable findings, succinic acid and D-mannose showed positive correlations with several pro-inflammatory cytokines, including TNF-α, IL-6, IL-1β, and MCP-1, suggesting that these metabolites may be involved in modulating inflammatory signaling in the intrauterine environment. Similarly, lactic acid, cholesterol, and glucose were positively correlated with multiple interleukins and chemokines, reinforcing their possible role in fueling immune cell activation and energy metabolism under suboptimal nutritional conditions.

Conversely, stearic acid and 2-palmitoylglycerol exhibited inverse correlations with key cytokines such as IL-1β, IL-17E, and IL-10, indicating a potentially anti-inflammatory or immunomodulatory effect of these lipids.

These exploratory correlations suggest possible associations between candidate metabolic alterations and the intra-amniotic inflammatory environment, but they do not establish mechanistic links or causal relationships. Such interactions may contribute to early programming of disease susceptibility, particularly in contexts such as asthma or metabolic syndrome.

## 4. Discussion

This exploratory study suggests an association between maternal nutritional adequacy during pregnancy and differences in the amniotic-fluid metabolomic profile during the second trimester. Although these findings are biologically plausible in the context of fetal programming, the observational and cross-sectional design does not allow causal inference.

Through untargeted metabolomic analysis, multivariate statistical modeling, pathway enrichment, and cytokine–metabolite correlation analysis, we explored possible associations between maternal nutritional adherence, the amniotic-fluid metabolomic profile, and the intra-amniotic inflammatory milieu.

### 4.1. Metabolomic Signatures Reflecting Nutritional Status

The stratification of the cohort using the FIGO Nutrition Checklist revealed significant differences in the amniotic fluid metabolome between women with adequate versus inadequate nutritional adherence. Importantly, PLS-DA analysis suggested partial separation between the two nutritional groups. However, because of the small and imbalanced cohort, this finding should not be interpreted as confirmatory evidence of a stable discriminant model. Rather, it indicates a potential association between maternal nutritional adequacy and the amniotic-fluid metabolomic profile, which should be validated in larger independent cohorts.

These findings echo previous reports suggesting that the amniotic fluid metabolome is highly responsive to maternal lifestyle and nutritional status [8,16].

Among the most discriminatory metabolites, fructose, 3-ketovaleric acid, and fructose-1,6-bisphosphate (Figure 1B) were more abundant in women with poor dietary adherence. These metabolites are related to glycolysis and energy metabolism and may be compatible with differences in energy-related biochemical profiles between groups. However, metabolite abundance alone does not allow direct inference of pathway activity or impaired carbohydrate regulation. Elevated glucose and related metabolites in the amniotic fluid have been associated with gestational diabetes and increased fetal insulin production, predisposing offspring to macrosomia and insulin resistance later in life [11].

Additionally, lipid-related metabolites such as myristic acid and stearic acid were differentially represented. While saturated fatty acids like stearic acid are commonly associated with metabolic disorders, their presence in AF may reflect complex fetal lipid utilization patterns rather than simple dietary transfer. Interestingly, stearic acid was inversely correlated with several inflammatory cytokines (Figure 3), suggesting a possible immunomodulatory function within the fetal milieu, consistent with emerging roles of fatty acids in immune regulation [17].

It should also be noted that the dichotomization of the FIGO Nutrition Checklist score was used as an exploratory analytical strategy. Although useful for comparing metabolomic profiles between groups, this binary classification may oversimplify the continuous nature of maternal dietary adherence and should not be considered a diagnostic classification of nutritional inadequacy.

### 4.2. Metabolic Pathways Implicated in Fetal Programming

The exploratory pathway enrichment analysis provided a preliminary biological framework for interpreting the candidate metabolites associated with lower nutritional adherence. The highest-ranking pathways involved amino-acid metabolism, energy metabolism, and lipid-related processes. However, these pathways did not remain statistically significant after Holm correction and should therefore be considered hypothesis-generating. Their biological plausibility is consistent with known mechanisms involved in fetal metabolic adaptation, but larger and adequately powered studies are required to confirm whether these pathways are reproducibly associated with maternal nutritional status [1,3].

Of particular interest is the enrichment of arginine and proline metabolism, given the role of proline in collagen synthesis and neurodevelopment. Elevated proline levels in Group 2 may reflect fetal adaptations to oxidative stress or placental insufficiency—both mechanisms previously implicated in adverse perinatal outcomes and neurodevelopmental disorders [16].

### 4.3. Immunometabolic Cross-Talk in the Intrauterine Environment

The integration of cytokine profiling with metabolomic data was intended to provide an exploratory view of possible associations between the amniotic-fluid metabolic profile and the intra-amniotic inflammatory milieu. Because cytokine analysis was performed in a subset of samples and involved multiple metabolite–cytokine correlations, these results should be interpreted with particular caution. After correction for multiple testing, the correlation heatmap was considered hypothesis-generating rather than confirmatory evidence of immunometabolic cross-talk [18].

Lactic acid, a marker of anaerobic metabolism and hypoxia, also correlated with elevated cytokine levels. This suggests possible metabolic reprogramming of the fetus in response to a less favorable intrauterine environment. Lactic acidosis has been observed in fetuses from pregnancies complicated by gestational diabetes and preeclampsia, and may reflect mitochondrial dysfunction or energy deficits [11].

Conversely, stearic acid and 2-palmitoylglycerol were negatively associated with several cytokines, including IL-1β, IL-17E, and IL-10. These lipids may exert anti-inflammatory effects, either directly or by modulating membrane composition and receptor signaling. Such patterns highlight the possibility that some fetal metabolites act as compensatory mechanisms to dampen inflammation under metabolic stress.

### 4.4. Clinical Implications and Future Directions

These preliminary findings may have implications for future research in maternal-fetal medicine, particularly for the development of biochemical approaches aimed at characterizing the intrauterine environment. However, the potential role of prenatal biomarkers in risk stratification should be interpreted in relation to clinically measurable pregnancy and neonatal outcomes, as previously shown in studies linking first-trimester screening parameters with neonatal complications [19]. In the present study, no longitudinal neonatal follow-up was available; therefore, the observed metabolomic differences should not be interpreted as predictive biomarkers but rather as candidate features requiring validation.

Amniotic-fluid metabolomics may provide a useful approach for characterizing the fetal biochemical environment during pregnancy, although its clinical relevance requires validation in longitudinal outcome-based studies. These exploratory findings may help generate hypotheses for future studies evaluating whether maternal nutritional patterns are associated with measurable changes in the intrauterine biochemical environment and with clinically relevant pregnancy or neonatal outcomes [2].

In light of these results, longitudinal studies are warranted to assess whether the metabolic profiles identified here persist in neonatal blood or urine, and whether they correlate with outcomes such as birth weight, early growth trajectories, and childhood immune or metabolic disorders. In particular, asthma and obesity, two of the most frequently studied DOHaD-linked conditions, have been associated with altered metabolomic and immune profiles both prenatally and postnatally [20].

Furthermore, the integration of multi-omics approaches, including epigenomics and microbiomics, could help identify mechanistic links between maternal environment, fetal metabolism, and postnatal health.

### 4.5. Limitations

The principal limitation of this study is the limited sample size, particularly the small number of participants in the inadequate nutrition group. This imbalance reduces statistical power and may increase the risk of unstable estimates, random findings, and overfitting in supervised multivariate models. For this reason, the metabolomic findings should be interpreted as exploratory and hypothesis-generating. Larger, balanced, and independent cohorts are required to validate these preliminary results and to evaluate their potential clinical relevance.

The cross-sectional nature of the data also precludes causal inference.

Cytokine profiling was performed in 37 of the 41 available amniotic-fluid samples, including 28 samples from the FIGO >5 group and all 9 samples from the FIGO ≤5 group. Although the lower-adherence group was fully represented, cytokine data were not available for the entire cohort; therefore, cytokine–metabolite correlation analyses should be considered exploratory and require confirmation in larger cohorts with complete cytokine coverage.

Moreover, because all participants were recruited among women undergoing clinically indicated amniocentesis, the cohort may not fully represent the general pregnant population. Although the indications for amniocentesis were recorded and compared where available, referral-related selection bias cannot be completely excluded.

Although BMI and gestational weight gain did not differ significantly between groups, both tended to be higher in the lower nutritional adherence group and may represent clinically relevant confounders in a small cohort. Because of the limited sample size, particularly the small number of participants in the lower adherence group, multivariable adjustment for maternal adiposity-related variables was not performed, and residual confounding by BMI or gestational weight gain cannot be excluded.

Another limitation is that nutritional status was dichotomized using a FIGO Nutrition Checklist threshold that was applied for exploratory stratification and should not be interpreted as an externally validated diagnostic cut-off.

Pathway enrichment results should also be interpreted cautiously, as Holm-adjusted *p*-values did not reach conventional statistical significance; therefore, these analyses are exploratory and should be validated in larger independent datasets.

## 5. Conclusions

This exploratory study suggests that maternal nutritional adequacy during pregnancy is associated with differences in the amniotic-fluid metabolomic profile during the second trimester. These preliminary findings may contribute to the understanding of the fetal biochemical environment in relation to maternal nutritional status. However, because of the observational design, limited sample size, and imbalance between groups, no causal inference can be made. Larger, balanced, and longitudinal studies integrating maternal, fetal, and neonatal outcomes are required to validate these associations and clarify their potential clinical relevance.

## Figures and Tables

**Table 1:** Demographic and anthropometric characteristics of enrolled patients.

Parameter	Higher Nutritional Adherence, FIGO > 5, *n* = 32	Lower Nutritional Adherence, FIGO ≤ 5, *n* = 9
Maternal age (years)	33.46 ± 5.95	35.50 ± 7.13
Pre-pregnancy weight (kg)	64.60 ± 14.09	71.14 ± 15.56
Current weight (kg)	69.54 ± 15.36	76.58 ± 14.87
Pre-pregnancy BMI (kg/m^2^)	24.22 ± 4.54	27.00 ± 5.50
Current BMI (kg/m^2^)	26.04 ± 4.79	29.31 ± 6.07
Gestational weight gain (kg)	5.29 ± 4.88	6.91 ± 3.61
Gestational age at amniocentesis (weeks)	17.89 ± 1.67	18.03 ± 2.21

## Data Availability

The datasets presented in this article are not readily available, as the data are part of an ongoing study. Partial data can be requested from the corresponding authors.

## Abbreviations

The following abbreviations are used in this manuscript:

AF	Amniotic Fluid
BMI	Body Mass Index
BSTFA	N,O-Bis(trimethylsilyl)trifluoroacetamide
DNA	Deoxyribonucleic Acid
DOHaD	Developmental Origins of Health and Disease
FIGO	International Federation of Gynecology and Obstetrics
GC-MS	Gas Chromatography–Mass Spectrometry
HMDB	Human Metabolome Database
IL	Interleukin
KEGG	Kyoto Encyclopedia of Genes and Genomes
LC-MS	Liquid Chromatography–Mass Spectrometry
LGA	Large for Gestational Age
MCP-1	Monocyte Chemoattractant Protein-1
PCA	Principal Component Analysis
PBMC	Peripheral Blood Mononuclear Cells
PLS-DA	Partial Least Squares–Discriminant Analysis
RNA	Ribonucleic Acid
TNF-α	Tumor Necrosis Factor-alpha
VIP	Variable Importance in Projection
